# Highly Elastic, Bioresorbable Polymeric Materials for Stretchable, Transient Electronic Systems

**DOI:** 10.1007/s40820-023-01268-2

**Published:** 2024-02-01

**Authors:** Jeong-Woong Shin, Dong-Je Kim, Tae-Min Jang, Won Bae Han, Joong Hoon Lee, Gwan-Jin Ko, Seung Min Yang, Kaveti Rajaram, Sungkeun Han, Heeseok Kang, Jun Hyeon Lim, Chan-Hwi Eom, Amay J. Bandodkar, Hanul Min, Suk-Won Hwang

**Affiliations:** 1 KU-KIST Graduate School of Converging Science and Technology, Korea University, 145 Anam-ro, Seongbuk-gu, Seoul, 02841 Republic of Korea; 2 SK Hynix, 2091, Gyeongchung-daero, Bubal-eup, Icheon-si, Gyeonggi-do 17336 Republic of Korea; 3 Hanwha Systems Co., Ltd., 188, Pangyoyeok-ro, Bundang-gu, Seongnam-si, Gyeonggi-do 13524 Republic of Korea; 4 Department of Electrical and Computer Engineering, North Carolina State University, Raleigh, NC 27606 USA; 5 Center for Advanced Self-Powered Systems of Integrated Sensors and Technologies (ASSIST), North Carolina State University, Raleigh, NC 27606 USA; 6 Department of Integrative Energy Engineering, Korea University, 145 Anam-ro, Seongbuk-gu, Seoul, 02841 Republic of Korea; 7 Biomaterials Research Center, Korea Institute of Science and Technology (KIST), 5 Hwarang-ro 14-gil, Seongbuk-gu, Seoul, 02792 Republic of Korea; 8 Semiconductor R&D Center, Samsung Electronics Co., Ltd., Hwaseong-si, Gyeonggi-do 18448 Republic of Korea; 9 Center for Advanced Biomolecular Recognition, Biomedical Research Division, Korea Institute of Science and Technology (KIST), Seoul, 02792 Republic of Korea

**Keywords:** Biodegradable elastomer, Conductive polymer composites, Biomedical device, Transient electronics

## Abstract

Substrates or encapsulants in soft and stretchable formats are key components for transient, bioresorbable electronic systems; however, elastomeric polymers with desired mechanical and biochemical properties are very limited compared to non-transient counterparts. Here, we introduce a bioresorbable elastomer, poly(glycolide-*co-ε*-caprolactone) (PGCL), that contains excellent material properties including high elongation-at-break (< 1300%), resilience and toughness, and tunable dissolution behaviors. Exploitation of PGCLs as polymer matrices, in combination with conducing polymers, yields stretchable, conductive composites for degradable interconnects, sensors, and actuators, which can reliably function under external strains. Integration of device components with wireless modules demonstrates elastic, transient electronic suture system with on-demand drug delivery for rapid recovery of post-surgical wounds in soft, time-dynamic tissues.

**Supplementary Information:**

The online version contains supplementary material available at 10.1007/s40820-023-01268-2.

## Highlights

- The paper introduces a bioresorbable elastomer, poly(glycolide-*co*-*ε*-caprolactone) (PGCL), with remarkable mechanical properties, including high elongation-at-break (< 1300%), resilience, and toughness (75 MJ m^−3^) for soft and transient electronics.
- Fabrication of conducting polymers with PGCL yields stretchable, conductive composites for transient electronic devices, functioning reliably under external strains.
- The study demonstrates the feasibility of a disintegrable electronic suture system with on-demand drug delivery for rapid recovery of post-surgical wounds on soft, time-dynamic tissues or versatile biomedical areas of interest.

**Supplementary Information:**

The online version contains supplementary material available at 10.1007/s40820-023-01268-2.

## Graphical abstract

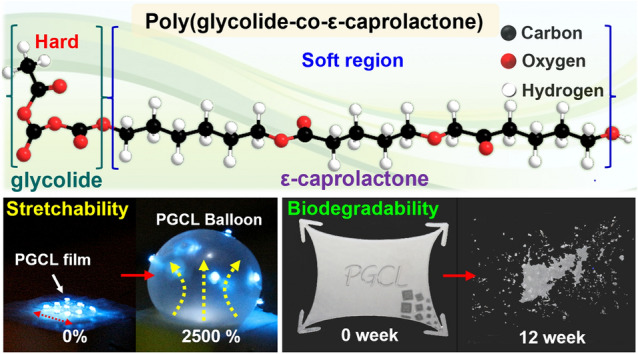

## Introduction

Electronic systems with the ability to physically dissolve or decompose after intended periods of use, sometimes referred to as ‘transient electronics,’ have opened-up unprecedented opportunities in diverse research areas, including temporary biomedical devices [1–6], drug delivery carriers [7, 8], disposable wearable components [9, 10], eco-friendly electronics [11, 12], and hardware security systems [13, 14]. These devices have been typically constructed with degradable and bio-safe polymers as substrates and encapsulation layers [15–17]. Examples include poly(lactide-*co*-glycolide) (PLGA) [9, 18], polylactide (PLA) [7, 18], silk fibroin [19, 20], and cellulose [21, 22] as natural, synthetic biodegradable polymers. However, inherent lack of elasticity led to challenges not only in harnessing the potential of transient electronics on mechanically dynamic biological tissues and organs, but also in extending their applications to highly deformable and advanced electronic systems, e.g., epidermal electronics [23, 24], soft robotics [25, 26], and human–machine interfaces [9, 27–32]. In an effort to address the absence of the key material, recent researches introduced man-made or nature-derived dissolvable stretchable polymers such as poly(lactide-*co*-*ε*-caprolactone) (PLCL) [33], polyurethanes (PU) [34, 35], and citric acid, gelatin, or polyethylene glycol (PEG)-based synthetic polymers [25, 36–38]. Nonetheless, only few of these materials exhibited a desirable range of mechanical, physical, and biochemical properties for practical, extensive uses. In this context, further development and discovery of biodegradable elastomers with the desired characteristics could broaden the range of material options for transient and dissolvable electronics.

In the following, we introduce a highly stretchable, bioresorbable polyester, poly(glycolide-*co*-*ε*-caprolactone) (PGCL), which has been restrictively used in surgical sutures, stents, and tissue engineering [39–42]. Comprehensive examinations of materials provide diverse ranges of characteristics of PGCLs, and assembly with a conducting polymer creates conductive elastomeric composites for stretchable interconnects and thermal actuators. Demonstrated platform suggests an elastic, disintegrable electronic suture system with drug elution in a wireless mode to accelerate the healing process of surgical wounds, particularly in soft, time-dynamic tissues.

## Experimental Section

### Evaluation of Physical Properties of PGCLs

About 10 wt% solutions of PGCL (PGCL 70:30, PGCL 55:45, and PGCL 15:85, Akina Inc., USA) dissolved in dimethylformamide (DMF, Sigma-Aldrich, USA) were poured onto polydimethylsiloxane (PDMS, Sylgard 184, Dow Corning, USA) molds with shapes corresponding to the ASTM D1708-18 standard test method. The solutions were fully evaporated at 60 °C and then dried in an oven at 25 °C for 2 days, resulting in dumbbell-shaped specimens with a thickness of 0.2 mm. Mechanical properties were examined using a universal testing machine (Instron 8801, Intron) at a crosshead displacement rate of 10 mm min^−1^ and at room temperature. The ductile behavior of PGCL polymers was assessed by performing repeated load–unload (0–50%) tensile tests under the same environmental conditions.

### Degradation Characteristics of PGCLs

PGCL polymers (250-μm thick) were prepared and cut into 1 × 1 cm^2^ sizes. Conical tubes with a volume of 50 mL were filled with numerous 0.1 M PBS solutions ranging from acidic to basic (pH 1–pH 13). The polymer films were fully immersed in each solution at 37 °C (physiological condition) to measure the weight change of the degraded films over time in a dried state. Additionally, PGCL films were fabricated to examine the degradation of tensile strength with immersion time, following the ASTM D1708-18 specification. Mechanical loading tests were conducted on fully dried PGCL specimens after removing the film from the PBS solution (pH 7) at 37 °C, performed weekly.

### Elastomeric Encapsulant Demonstration of PGCLs

Silicon (Si) wafers were initially prepared with a coating of poly(methyl methacrylate) (PMMA) and polyimide (PI) as temporary carrier substrates. A layer of magnesium (Mg), approximately 1-μm thick, was deposited using e-beam evaporation and subsequently patterned. Inorganic encapsulants (SiO_2_, Si_3_N_4_) were then applied via plasma-enhanced chemical vapor deposition (PECVD). Following this, a wet-etch process employing buffered oxide etching (BOE) solution was utilized to create metal pads for measurements, and a dry etcher was employed to define the magnesium device using oxygen. The device was then transferred from the carrier wafer to a temporary stamp made of PDMS by etching away the sacrificial layer and exposing it to acetone solvent. Subsequently, the bottom polyimide layer was removed using a dry etcher, and the device was placed onto a layer of PGCL polymers. Fabrication process of Mg device finished with the application of a top PGCL layer, followed by drying. For the encapsulation test, the device was affixed to a uniaxial stretcher, and PDMS mold was secured onto the device using epoxy. The mold was then filled with water, and changes in resistance were measured at regular intervals.

### Synthesis and Characterization of the Degradable, Conductive, and Elastic Composite

PEDOT:PSS aqueous solution (PH1000, Clevious, USA) was subjected to liquid nitrogen (N_2_) for 10 min and then placed under vacuum conditions (5 mTorr) to lyophilize at − 85 °C for 24 h. The resulting solid (1 wt%) was dissolved in dimethyl sulfoxide (DMSO, Sigma-Aldrich, USA) solvent for 2 h using an ultrasonic processor (VCX-500, Sonics and Materials, USA). Different ratios of 1 wt% PGCL (55:45) solution (in DMSO solvent) and d-sorbitol were mixed with the PEDOT:PSS solution to evaluate the changes in mechanical and electrical performance of the conductive composite. The composite solutions were drop-cast onto a PDMS mold and dried in an oven at 60 °C for 2 days to create conductive PGCL films. The conductivity of the composite was measured using a 4-point probe system (Keithley 2400 digital multimeter, Keithley). The stretchability of the composite was evaluated using a universal testing machine (Instron 8801, Intron).

### A Conductive PGCL Composite Heaters

PGCL (55:45) substrates were prepared by solution casting a 10 wt% PGCL (55:45) solution in DMF onto a PDMS mold, followed by solvent evaporation at 60 °C for 24 h. The optimized conductive composite solution (PGCL 55:45: PEDOT:PSS = 4:6 weight ratio, d-sorbitol: 2% w/w of PEDOT:PSS) was drop-cast onto the PGCL substrate, and the resulting layers were dried at 60 °C for 24 h to form the conductive heating layers. The conductive PGCL composite heaters were then encapsulated with PGCL (55:45), and the solvent was removed to complete the fabrication process.

### Drug-Eluting PGCL Thread

Negative molds with a semicylinder rod shape and a 0.5-mm radius were created using a 3D printer (Cr-10s, Creality 3D). A PDMS (10:1) solution was then filled into the printed mold and cured at 60 °C for 24 h to produce the PDMS mold. Ketorolac tromethamine salt, a nonsteroidal anti-inflammatory drug (50 mg), was dissolved in DMSO (10 mL) to create a drug solution. PGCL (15:85) (5 g) was added to 10 mL of the drug solution and magnetically stirred at 60 °C for 1 day. The viscous PGCL mixture solution (50 wt%) was carefully pipetted into the mold, and the solvent was thoroughly evaporated in a nitrogen-purged oven for 2 days to fabricate the drug-eluting PGCL suture. The loading capacity of drugs in PGCL can be regulated based on factors such as the type of drug, the subject, and the therapeutic procedure [43–45].

### Fabrication of MED-Suture

First, a sacrificial layer consisting of PMMA/PI was spin-coated onto a carrier wafer. Next, photolithographic patterning of deposited tungsten (W)/Mg layers was performed to define the interconnect (100-nm/1-μm thick) and temperature sensor (10-/100-nm thick) using an e-beam evaporating system and sputter. The device was then transferred from the carrier wafer to a temporary PDMS stamp by etching the sacrificial layer and exposing it to acetone solvent. Subsequently, bottom polyimide layer was removed using a dry etcher, and the device was placed onto a layer of PGCL (55:45)/PGCL heater/PGCL (55:45) (~ 50-μm thick). The thread was precisely patterned with a width of 0.8 mm using a laser-cutting system. Electronic thread layer was laminated onto the bottom drug-eluting PGCL suture (semicylinder), followed by the addition of another heater layer and the top drug-eluting PGCL suture (semicylinder) on top of the electronic thread layer. Finally, multiple layers were compacted through hot pressing with cylinder-shaped mold, resulting in a fully bioresorbable smart suture (cylinder).

### Design and Fabrication for Wireless Module

A biodegradable magnesium (Mg) foil (20-μm thick) was attached to a PDMS-coated glass substrate. A layer of tungsten (W) (100-nm thick) was sputter-coated onto the metal foil, and laser lithography was used to define the receiving inductive coil, interconnects, and contact pads for connecting the commercially packaged electronic components. Oxygen plasma pre-treatment was applied to improve the tackiness of both the metallic devices and the PGCL (55:45) substrate (150-μm thick) to facilitate transfer printing from a PDMS stamp to the PGCL substrate. Encapsulation process involved covering the laser-patterned PGCL (55:45) film (50-μm thick) with via holes (top layer) and hot pressing. The wireless module of the smart suture included various components such as a bluetooth low-energy system-on-chip (nRF52832), amplifier (INA2321), low drop-out regulator (LM1117), boost DC/DC converter (LT1615), diode, and capacitor. All electrical components were connected using conductive epoxy, and the electronic suture was electrically bonded through a via hole in the bottom substrate.

## Results and Discussion

### Mechanically Resilient, Bioresorbable Elastomer PGCL

**Fig. 1:**
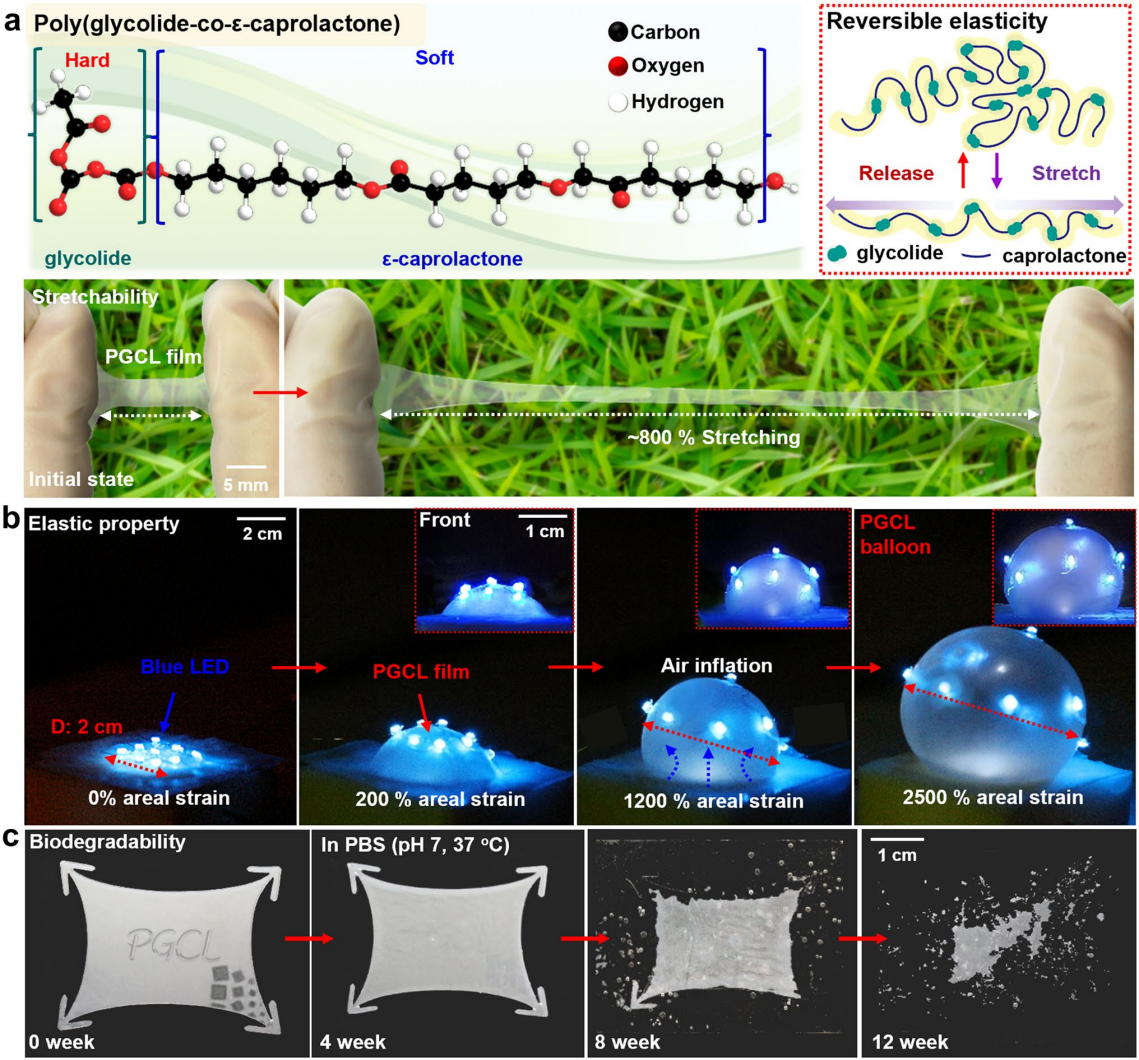
Stretchable, biodegradable elastomeric polymer. **a** Chemical structure of stretchable and biodegradable elastomer, poly (glycolide-co-ε-caprolactone) (PGCL). The copolymer consists of glycolide as a hard, cross-linker segment and ε-caprolactone as a soft, elastic domain (top). Extreme stretchability up to ~800% of a PGCL film (150 μm thick), with an image at the initial state (bottom). **b** A set of images showing superior elasticity through a wide range of areal strains (~2500%) using inflation of PGCL balloons combined with light-emitting diodes (LEDs). **c** Time-sequential degradation images of a PGCL substrate (thickness: 250 μm) in phosphate buffer saline (PBS, pH 7) solution at body temperature (37 °C) during 12 weeks

Figure 1a exhibits molecular structure of a highly stretchable and biologically degradable elastomer, PGCL, produced via physical crosslinking of glycolide or glycolic acid (GA) with ε-caprolactone (CL) by ring-opening polymerization [40, 41], which GA and CL are responsible for hard and soft segments, respectively. Unlike stiff and inelastic homopolymers of GA and CL [i.e., polyglycolic acid (PGA) and polycaprolactone (PCL)] [46, 47], the heterogeneous mixtures with various ratios of GA to CL present a wide range of rubber-like stretchable property. As an example, PGCL (55:45) can withstand an uniaxial tensile strain over 800%. Figure 1b exhibits a set of images of mechanically pliable PGCL balloons (thickness, ~ 150 μm) integrated with an array of blue light-emitting diodes (LEDs) (other images appear in Fig. S1), and the inflated PGCL balloon with superior stretchability expanded up to ~ 2500% areal strain without physical failures. Figure 1c presents degradation behavior of PGCL (~ 250-μm thick) during immersion in a phosphate buffer saline (PBS, pH 7) at physiological temperature (37 °C). The hydrolytically vulnerable ester bonds (–COO–) in the PGCL broke into aliphatic alcohols (–OH) and carboxyl acids (–COOH), which is the well-known decomposition mechanism of PGCL in water [40, 41]. As a result, PGCL films gradually disappeared during 12 weeks, and such behaviors were comparable to those reported in the previous articles [40, 41, 48].

### Comprehensive Characterizations of Bioresorbable Elastomer for Transient Electronics

**Fig. 2:**
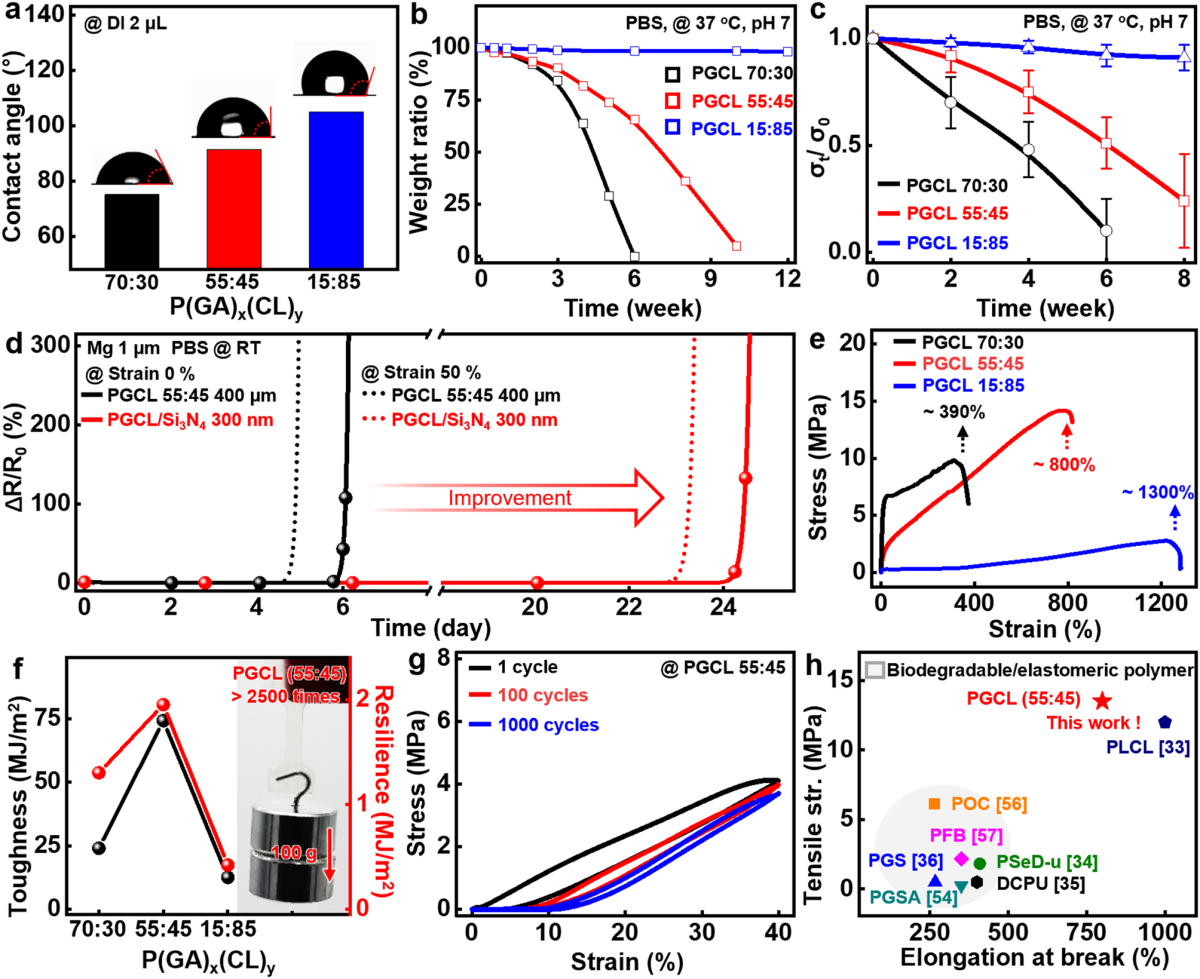
Comprehensive characteristics of PGCL polymers with different compositions. **a** Measured wettability of different PGCL polymers using deionized (DI) water droplets (2 μL). **b** Dissolution behaviors via hydrolysis and **c** changes in tensile strength with immersion time of the polymers in PBS solution (pH 7) at 37 °C. **d** Resistance profiles of Mg electrodes (thickness: 1 μm) encapsulated with a layer of stretchable PGCL (~ 400 μm), layers of and PGCL/Si_3_N_4_ (Si_3_N_4_ thickness, 300 nm), upon contact with PBS solution at room temperature (RT). **e** Stress–strain curves of PGCL films (150 μm) with different compositions (black, 70:30; red, 55:45; and blue, 15:85). **f** Mechanical properties of the copolymers depending on the ratio of glycolide (GA) and caprolactone (CL). **g** Changes in a mechanical behavior of PGCL films (thickness: 150 μm) under cyclic stretching tests at 40% strain. **h** Comparison of mechanical performances with previously reported biodegradable elastomeric materials [33–36, 54, 56, 57]

Dissolution rates of PGCL are closely related to attraction or repulsion to water. Figure 2a exhibits the wettability of PGCL with various compositions of GA and CL, determined by contact angles with deionized (DI) water. For a high content of CL, the elastomeric polymer indicated hydrophobic properties due to the alkyl chain (R–CH_2_–) of CL that is non-polar in nature, to hinder attraction of water molecules to the matrix [48, 49]. On the other hand, the elastomer with a high GA amount showed the opposite phenomenon. Schematic illustration of degradation mechanism of PGCL polymers appears in Fig. S2. Figure 2b presents measured dissolution rates of different PGCL polymers (~ 150-μm thick) while immersed in PBS (pH 7) at 37 °C, and degradation occurred in a very slow fashion as the CL content increased. Water uptake and pH effects of the solution on the polymers appear in Fig. S3. Similar tendency was observed in changes of tensile strength (*σ*) on dissolution (Fig. 2c). When immersed in PBS (pH 7) at 37 °C, both PGCLs (70:30 and 55:45) lost 50% of mechanical strengths in 4 and 6 weeks, respectively. However, PGCL (15:85) retained the mechanical property over 8 weeks, which shows potential applicability as substrate/encapsulation of transient electronics that need to operate for a long period of time. Degradation characteristics of PGCL exhibit a relatively slower degradation rate compared to other reported biodegradable polymers including PLGA (~ 6 weeks) [50, 51], silk fibroin (35 wt% loss in 3 weeks) [52], and poly(sebacoyl diglyceride)-based elastomer (60 wt% loss within 8 h) [34], and biodegradable hydrogel including gelatin methacrylate (GelMa) (~ 10 days) [53], PEG-based adhesive hydrogel (~ 20 days) [38], and poly(glyceryl-sebacate-acrylate) (PGSA) (90 wt% loss in 8 weeks) [54]. Unlike those polymers, PGCL has the ability to control degradation behavior and mechanical elasticity in various ways by adjusting the composition of GA and CL, which provide diverse options for soft, biodegradable electronics.

Figures 2d and S4 represent measured changes in resistance (R) of magnesium resistors (Mg, 1-μm thick) encapsulated with PGCLs (55:45, 100–400-μm thick) and inorganic thin films such as silicon dioxide (SiO_2_, 300-nm thick) and silicon nitride (Si_3_N_4_, 300-nm thick), when contacted with PBS at room temperature (RT). Slight loss of performances under a 50% uniaxial strain was probably due to reduction of the thicknesses in the direction perpendicular to the tensile strain [55]. A single layer of PGCL yielded a lifetime of approximately 6 days while the assembled organic/inorganic bilayers increased the lifetime to ~ 22 and 24 days for SiO_2_ and Si_3_N_4_, respectively (detailed device structure and images appear in Fig. S4). This efficacy of the combined encapsulants was similar to reported values in the previous studies; however, PGCL’s exceptional elastic behavior, not observed in natural and synthetic degradable polymers (e.g., silk fibroin, gelatin, PLGA, PLA, PCL, and PGA), can provide practical shielding effects even under diverse modes of external deformation [15–17].

Mechanical properties of PGCL depend primarily on the compositions (i.e., a ratio of GA to CL). The GA-rich PGCL (70:30) showed high elastic modulus (*E*, ~ 100 MPa) and low elongation-at-break (~ 390%), while the CL-rich PGCL (15:85) was relatively softer (*E*, ~ 2.7 MPa; elongation-at-break, ~ 1300%) than the other ones, as shown in Fig. 2e. The elastomer (*E*: ~ 15 MPa) with a near-equivalent ratio of 55:45 could be stretched up to ~ 800%, and showed high toughness (up to ~ 75 MJ m^−3^) and excellent resilience (up to ~ 2 MJ m^−3^) (Figs. 2f and S5). Image in the inset demonstrated the capability of lifting up a weight (100 g) over ~ 2500 times heavier than PGCL membrane (38 mg). Figures 2g and S6 exhibit results of a low hysteresis (~ 10%) as confirmed in cyclic tests with a linear strain of 40%. Overall comparison with the previous degradable elastomers [33–36, 54, 56, 57] in Fig. 2h presents outstanding mechanical performance of PGCL, especially in tensile strength and toughness, which overcomes the limitations of existing materials and provides a strong option for relevant research fields. Additional thermal, optical, and chemical properties appear in Fig. S7.

### Degradable Conductive Elastomer

**Fig. 3:**
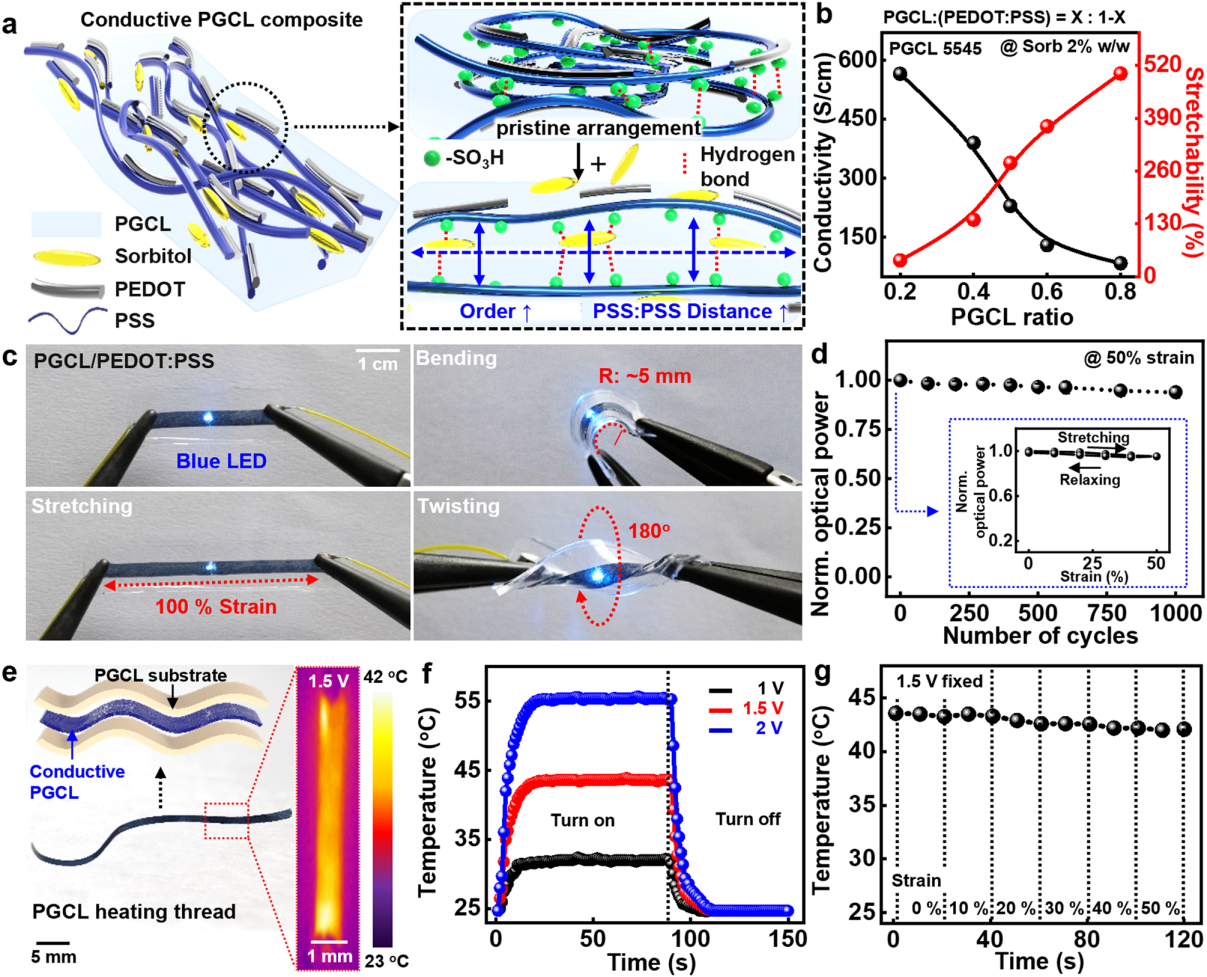
Degradable conductive elastomer. **a** Schematic illustration of an elastomeric conductive composite with poly(3,4-ethylenedioxythiophene):poly(styrenesulfonate) (PEDOT:PSS) as a conductor and d-sorbitol as an additive, with mechanisms of the modified structure in the inset. **b** Measurements of electrical and mechanical properties in various mixture ratios of PGCL and PEDOT:PSS at a constant concentration of d-sorbitol (2 w/w% of PEDOT:PSS). **c** Stable operation of the conductive composite connected with a blue LED, under external deformation modes, bending (top right), stretching (bottom left), and twisting (bottom right). **d** Measured light intensity of the blue LEDs during cyclic tests at a tensile strain of 50%, with the result of the first attempt in the inset. **e** Thread-like elastomeric heater, with infrared (IR) image of the device at an applied voltage of 1.5 V. **f** Temperature profiles of the heater at an applied voltage of 1–2 V. **g** Steady Joule heating performances of the elastic heater with stretching up to 50%

In addition to potential uses as substrate and encapsulation, PGCL provides an opportunity to fabricate conductive elastomeric composites when combined with conducting materials. Figure 3a illustrates a stretchable, disintegrable conductive composite: PGCL as a polymer matrix, poly(3,4-ethylenedioxythiophene)-poly(styrenesulfonate) (PEDOT:PSS) as a conductive filler, and d-sorbitol as a dopant. Six hydroxyl groups (–OH) of d-sorbitol interacted with sulfonate group (–SO_3_–) of PSS chain in PEDOT:PSS, which facilitated to separate conductive PEDOT-rich regions and insulating PSS-rich shell [58–60]. This phase separation induces untangled, linear conductive pathways of PEDOT, which remarkably improved electrical conductivity to a maximum value of ~ 600 S cm^−1^. Addition of d-sorbitol as a plasticizer softened the mechanical property (~ 1 MPa) and improved the stretchability up to ~ 560% [60]. Figure 3b presents the effect of a ratio of PGCL to PEDOT:PSS on electrical and mechanical behaviors. Details of other compositions, d-sorbitol, and electrical/mechanical tests appear in Figs. S8 and S9. A set of optical images in Fig. 3c show the PGCL composite as interconnects for stretchable devices. The integrated blue LED exhibits stable functions upon various deformation modes, such as bending (5 mm of radius), stretching (100% of strain), and twisting (180°). Cyclic stretching experiments with uniaxial strain of 50% resulted in negligible changes in luminance of the LED (Fig. 3d, and cyclic bending tests appear in Fig. S10), highlighting excellent electrical/mechanical properties of the composites. Application of the composite as strain sensing component appears in Fig. S11. As a different electronic tool, Fig. 3e represents a lightweight, flexible/stretchable thermal thread (width, 800 μm; thickness, 30 μm; and length, 8 cm) that can generate heat for thermotherapeutic electronics. The heating thread in the inset shows uniform temperature distribution in the whole region. Figure 3f shows temporal heating profiles at different input voltages of 1, 1.5, and 2 V. Heating performance presents that temperature value rapidly increased owing to its high conductivity, and reliably maintained the desired temperatures [61, 62]. Such thermal characteristics were not deteriorated by external mechanical loads (Fig. 3g), and repetitive on–off cyclic tests and effect of external strains appear in Fig. S12. Electrical behaviors of the conductive composites under PBS solution at 37 °C appear in Fig. S13.

### A Disintegrable, Elastic, and Electronic Suture System

**Fig. 4:**
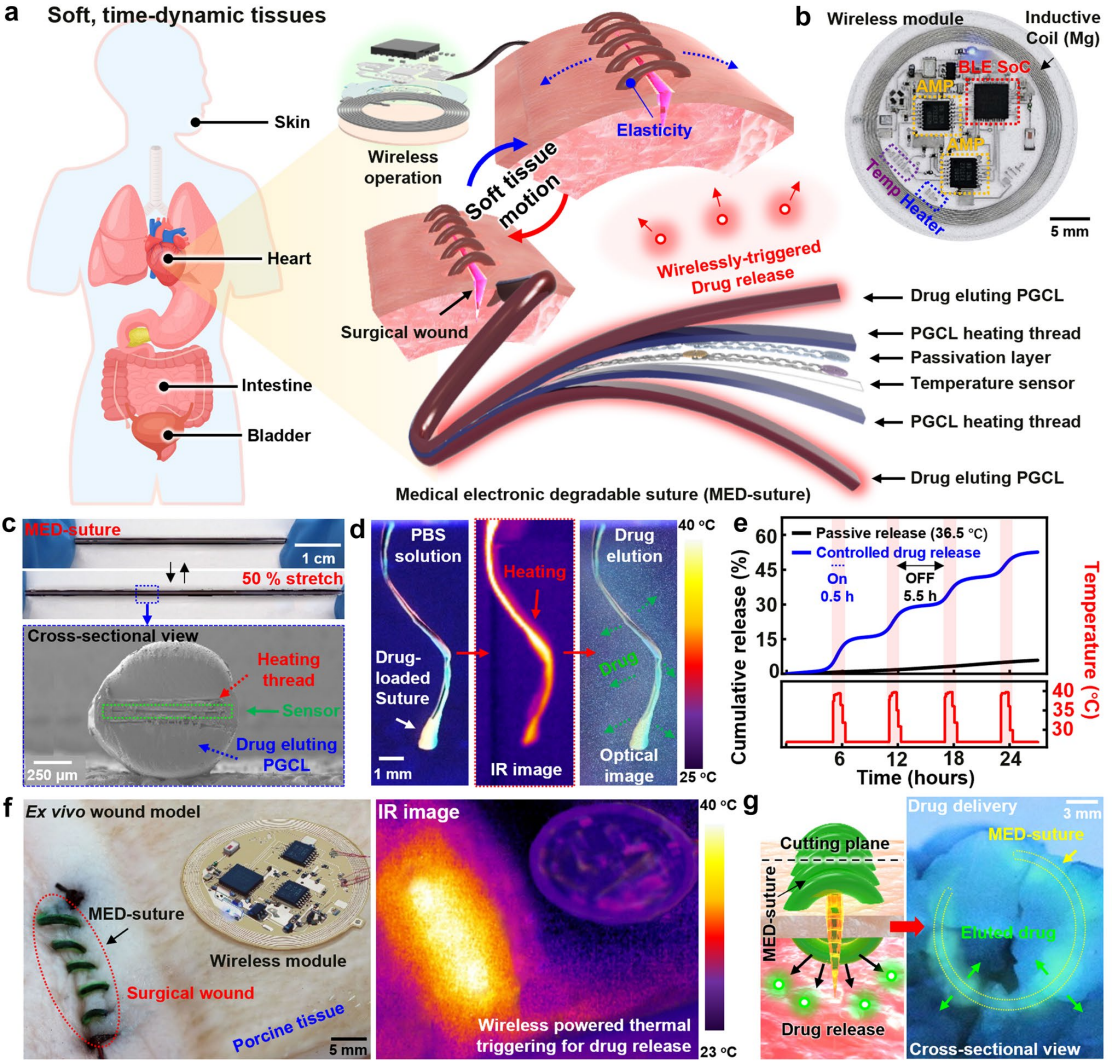
Transient electronic suture system. **a** Illustration of a medical, electronic, and degradable suture (MED-suture) system with a wireless circuit for surgical wound healing of post-operation sites on soft, time-dynamic tissues in the body. The electronic suture includes layers of on-demand drug vehicle (PGCL 15:85), conductive composite-based resistive heater (PGCL 55:45 composite), passivation layer (SiO_2_, 300 nm), electrical interconnects (W/Mg, 100-nm/1-μm thick), and temperature meter (W/Mg, 10-nm/100-nm thick). The wireless circuit includes microcontroller and bluetooth modules, amplifier, diodes, capacitors, and inductive coil (Mg, 20-μm thick). **b** Photograph of fabricated wireless system for MED-suture with multiple functions of temperature measurements and heating actuation. **c** Elastic performance of MED-suture with 50% of uniaxial tensile strain and cross-sectional view of its overall structure observed with scanning electron microscope (SEM). **d** In vitro drug delivery demonstration of MED-suture immersed in PBS solution at room temperature: (1) initial state (left), (2) thermal actuation (middle), and (3) drug elution (right). **e** On-demand drug release behaviors with ON/OFF cyclic thermal actuations and temperature monitoring profile for 24 h. **f** Ex vivo experiment setup for MED-suture using porcine skin with surgical incisions and IR images of heat-induced drug release via thermal actuation. **g** Experimental result after drug release on porcine tissues with four cyclic actuations

Figure 4a illustrates a medical, electronic, and degradable suture (MED-suture) connected with a wireless integrated circuit, particularly for time-dynamic biological tissues such as the heart, bladder, and intestine. The MED-suture secured the surgical wound and extended to the outer skin near the respective organ via dissolvable wires, to facilitate electrical connections with the removable wireless module for the on-demand treatment. The suture consists of a top drug-eluting layer (PGCL 15:85, ~ 400-μm thick), a top heating layer (PGCL 55:45/PEDOT:PSS, ~ 50 μm), a passivation layer (SiO_2_, 300 nm), a temperature sensor array (W/Mg, 10 nm/100 nm), metal interconnects (W/Mg, 100 nm/1 μm), a bottom heating layer (PGCL 55:45/PEDOT:PSS, ~ 50-μm thick), and a bottom drug-eluting layer (PGCL 15:85, ~ 400 μm). Detailed fabrication process and layer structure images appear in Figs. S14 and S15, respectively. Skin-attachable wireless modules included a microcontroller, a bluetooth system-on-chip (SoC), amplifiers, diodes, and a power receiver coil (Fig. 4b) and enabled diagnostic/therapeutic functions according to the operational block diagram (Fig. S16). Optical images (top) in Fig. 4c exhibit the elastic response of electronic suture threads under a uniaxial strain of 50%, and all the layers remained intact without delamination or failure, as observed in scanning electron microscopy (SEM) image (bottom). Such reliable elasticity allowed for consistent, stable temperature detection and thermal actuation under a stretching mode (50%) and even after 1000 cyclic bending tests at a radius of 2 mm (Fig. S17). The suture had an elastic modulus of ~ 5 MPa (Fig. S18), which is significantly lower than that of conventional suture threads [63, 64], however it is suitable for soft tissues [65–67].

Active drug release function in MED-suture plays a crucial role in the surgical wound healing process. The initial phase of wound is the most susceptible to severe inflammation and infection, potentially accompanied with acute pain or dangerous postoperative side effects. Actively releasing substantial amount of anti-inflammatory or antibacterial drug during this period would be an effective strategy rather than spontaneous slow release, to improve healing process [68–70]. Figure 4d, e captures images and data sets of thermo-responsive drug release behaviors of electronic sutures under physiological conditions. Upon thermal actuation to ~ 37 °C, an anti-inflammatory agent (ketorolac tromethamine, 1 wt%) with a green fluorescent dye (Alexa Fluor 488, 1 wt%, for visualization) was released due to increased chain movements of the drug-eluting matrix (PGCL 15:85; Tm, ~ 41 °C) (Fig. 4d) [7]. After four cycles of thermal actuation (on/off, 0.5/5.5 h for one cycle), ~ 50% of the total quantity of the drug was transported while a few percentile was released as passive diffusion under the same condition (Fig. 4e). Information of measurements and additional experiments appears in Fig. S19. We tested practical feasibility of the MED-suture using porcine skins with creation of wound sites (left), and corresponding IR image of thermally controlled threads via wireless power transmission (right) was shown in Fig. 4f. Repetitive, periodic thermal operations (on/off, 0.5/5.5 h) delivered the targeted drug to surgical sites or surrounding tissues, which was validated through apparent fluorescence on resected porcine models in Fig. 4g. In contrast, the control group (no thermal triggering) exhibited no observable fluorescence (Fig. S20).

## Conclusions

Materials, and device fabrication and integration presented here describe diverse features in a desirable manner of PGCL elastomers for versatile stretchable, transient electronics. A wide range of materials inspection provided properties of programmable degradation, excellent stretchability (< 1300%), and high toughness. Incorporation of a conductive polymer and surfactant into the polymer matrix produced transient, elastomeric conductive composites with outstanding electrical/mechanical properties, which enabled strain-tolerant functionality of interconnects and thermal actuators. Such materials with sophisticated device layouts assembled with wireless modules, created an elastic, disintegrable electronic suture system capable of wirelessly monitoring and facilitating the healing process of surgical wounds on soft and time-dynamic tissues.

## Supplementary Information

Below is the link to the electronic supplementary material.

Supplementary file1 (PDF 1518 KB)
