# Publisher Correction to: Highly Elastic, Bioresorbable Polymeric Materials for Stretchable, Transient Electronic Systems

**DOI:** 10.1007/s40820-024-01376-7

**Published:** 2024-03-06

**Authors:** Jeong-Woong Shin, Dong-Je Kim, Tae-Min Jang, Won Bae Han, Joong Hoon Lee, Gwan-Jin Ko, Seung Min Yang, Kaveti Rajaram, Sungkeun Han, Heeseok Kang, Jun Hyeon Lim, Chan-Hwi Eom, Amay J. Bandodkar, Hanul Min, Suk-Won Hwang

**Affiliations:** 1 KU-KIST Graduate School of Converging Science and Technology, Korea University, 145 Anam-ro, Seongbuk-gu, Seoul, 02841 Republic of Korea; 2 SK Hynix, 2091, Gyeongchung-daero, Bubal-eup, Icheon-si, Gyeonggi-do 17336 Republic of Korea; 3 Hanwha Systems Co., Ltd., 188, Pangyoyeok-ro, Bundang-gu, Seongnam-si, Gyeonggi-do 13524 Republic of Korea; 4 Department of Electrical and Computer Engineering, North Carolina State University, Raleigh, NC 27606 USA; 5 Center for Advanced Self-Powered Systems of Integrated Sensors and Technologies (ASSIST), North Carolina State University, Raleigh, NC 27606 USA; 6 Department of Integrative Energy Engineering, Korea University, 145 Anam-ro, Seongbuk-gu, Seoul, 02841 Republic of Korea; 7 Biomaterials Research Center, Korea Institute of Science and Technology (KIST), 5 Hwarang-ro 14-gil, Seongbuk-gu, Seoul, 02792 Republic of Korea; 8 Semiconductor R&D Center, Samsung Electronics Co., Ltd., Hwaseong-si, Gyeonggi-do 18448 Republic of Korea; 9 Center for Advanced Biomolecular Recognition, Biomedical Research Division, Korea Institute of Science and Technology (KIST), Seoul, 02792 Republic of Korea

**Publisher Correction to Nano-Micro Lett (2024) 16:102** 10.1007/s40820-023-01268-2

Due to typesetting mistake, Hanul Min was missed to be denoted as a corresponding author in the article. The typesetter apologizes for this.

The original article has been corrected.

