# Supplementary material for: Highly Elastic, Bioresorbable Polymeric Materials for Stretchable, Transient Electronic Systems
Source: Nanomicro Lett. 2024 Feb 1;16:102. doi: 10.1007/s40820-023-01268-2 (PMC10834929; doi:10.1007/s40820-023-01268-2)
Supplement: Supplementary file 1 — Supplementary file1 (PDF 1518 KB) [file 40820_2023_1268_MOESM1_ESM.pdf]

Supplementary Information for

Highly Elastic Bioresorbable Polymeric Materials for Stretchable Transient Electronic Systems

Jeong-Woong Shin[1], Dong-Je Kim[1], Tae-Min Jang[1], Won Bae Han[1], Joong Hoon Lee[1,2], Gwan-Jin Ko[1], Seung Min Yang[1,3], Kaveti Rajaram[4,5], Sungkeun Han[1], Heeseok Kang[1], Jun Hyeon Lim[1], Chan-Hwi Eom[1], Amay J. Bandodkar[4,5], and Suk-Won Hwang[1,6,7,*]

[1] KU-KIST Graduate School of Converging Science and Technology, Korea University, 145 Anam-ro, Seongbuk-gu, Seoul 02841, Republic of Korea

[2] SK Hynix, 2091, Gyeongchung-daero, Bubal-eup, Icheon-si, Gyeonggi-do 17336, Republic of Korea

[3] Hanwha Systems Co., Ltd. 188, Pangyoyeok-Ro, Bundang-Gu, Seongnam-si, Gyeonggi-do, 13524, Republic of Korea

[4] Department of Electrical and Computer Engineering, North Carolina State University, Raleigh, North Carolina 27606, United States

[5] Center for Advanced Self-Powered Systems of Integrated Sensors and Technologies (ASSIST), North Carolina State University, Raleigh, North Carolina 27606, United States

[6] Department of Integrative Energy Engineering, Korea University, 145 Anam-ro, Seongbuk-gu, Seoul, 02841, Republic of Korea

[7] Center for Biomaterials, Biomedical Research Institute, Korea Institute of Science and Technology (KIST), 5 Hwarang-ro 14-gil, Seongbuk-gu, Seoul, 02792, Republic of Korea

†Jeong-Woong Shin, Dong-Je Kim, Tae-Min Jang and Won Bae Han contributed equally to this work.

*Corresponding author. E-mail: dupong76@korea.ac.kr (Suk-Won Hwang)

Supplementary Figures

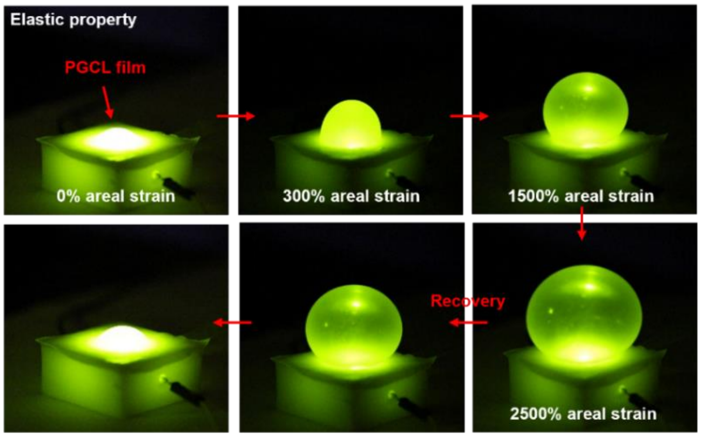

Fig. S1 Highly Expandable PGCL polymer. Sequential images of expansion test of PGCL (55:45) film (100 μm, thick) with pneumatic approaches

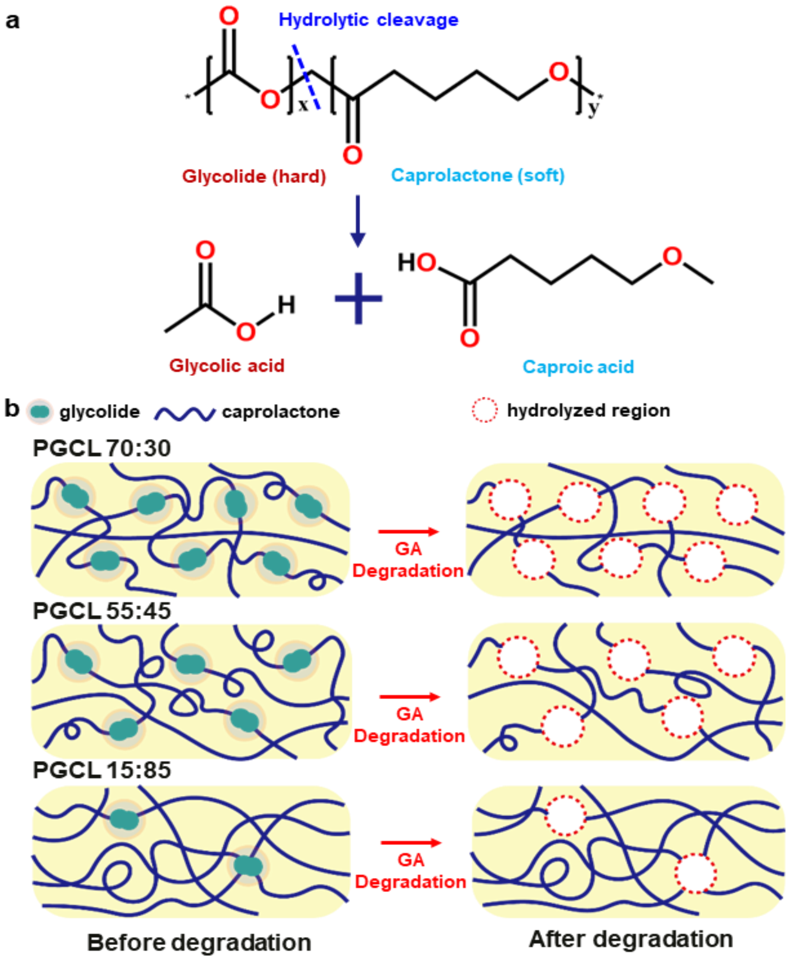

Fig. S2 Degradation mechanism of PGCL polymers with different contents. **a** Chemical reaction of hydrolysis of PGCL. **b** Schematic illustration of changes in polymer structure during degradation of GA segments

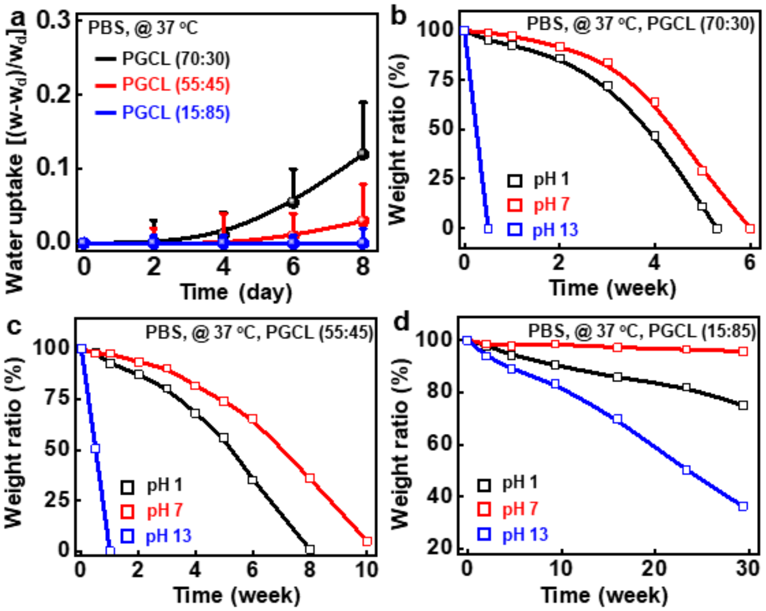

Fig. S3 Investigation of water uptake and pH effect of solution in degradation profiles with different PGCL films. a Water uptake profile of different PGCL films immersed in PBS solution at 37 °C. Changes of weight with respect to immersion time in acidic, neutral, and basic solutions: **b** PGCL (70:30), **c** PGCL (55:45), and **d** PGCL (15:85)

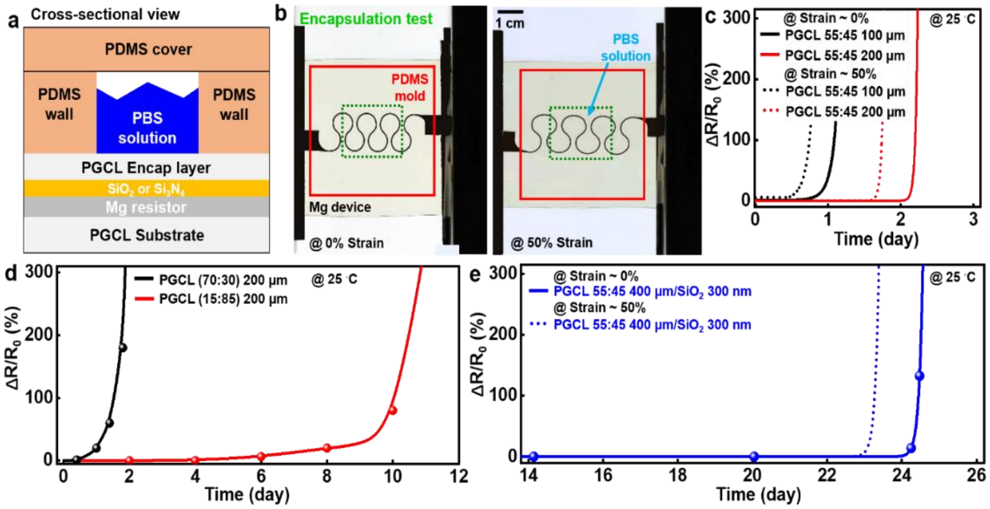

Fig. S4 Elastic, degradable encapsulant of PGCL film. **a** Cross-sectional illustration of encapsulation test set-up. **b** Encapsulation test of a serpentine-designed magnesium device in no applied strain (left) and applied 50% of strain (right) at room temperature. Each water reservoirs were attached on marked red square region. **c** Measurements of resistance changes of Mg device with different thickness of PGCL (55:45) films. **d** Encapsulation performances of different PGCL films. **e** Enhanced functional lifetime profiles of the device encapsulated by PGCL and SiO_2 (300 nm thick) passivation layer

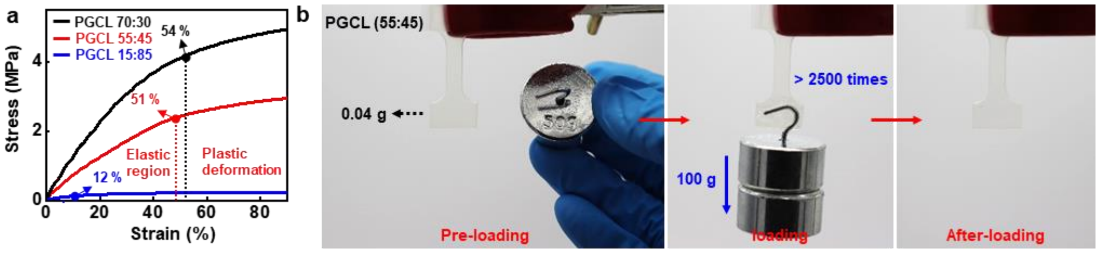

Fig. S5 Resilience and toughness of PGCL film. **a** Strain-stress curve in the initial condition of different PGCLs. **b** Toughness test images of PGCL (55:45) film loading with 100 g weights

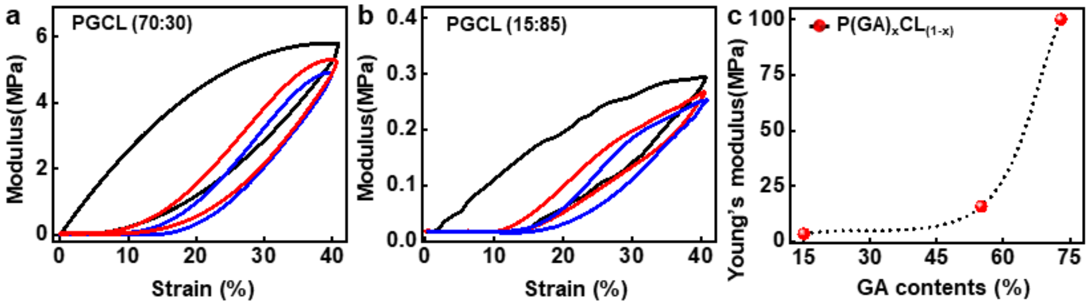

Fig. S6 Mechanical evaluation of PGCL films. Experimental results of cyclic tensile loading-unloading test of **a** PGCL (70:30) and **b** PGCL (15:85) films. **c** Calculated young's modulus with respect to glycolide contents in copolymers

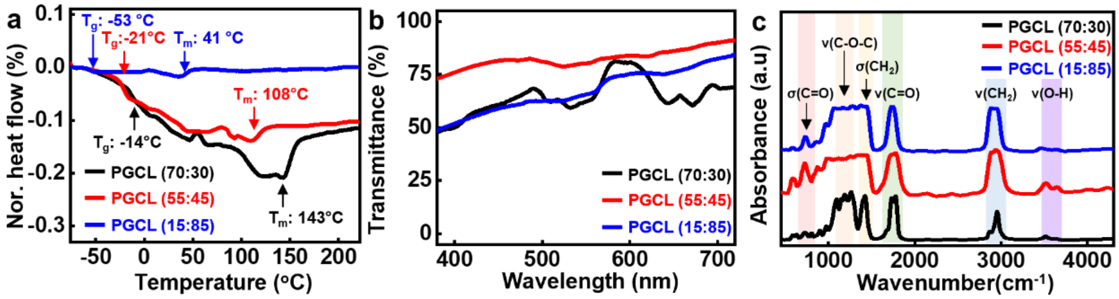

Fig. S7 Physical, optical, chemical characterization of PGCL films. **a** Thermal investigation of PGCL showing glass transition temperature and melting temperature of each PGCL films. **b** Transparency measurement of PGCL films in visible light region. **c** Infrared spectrum of PGCL polymers to analyse chemical bonding ratio in copolymers

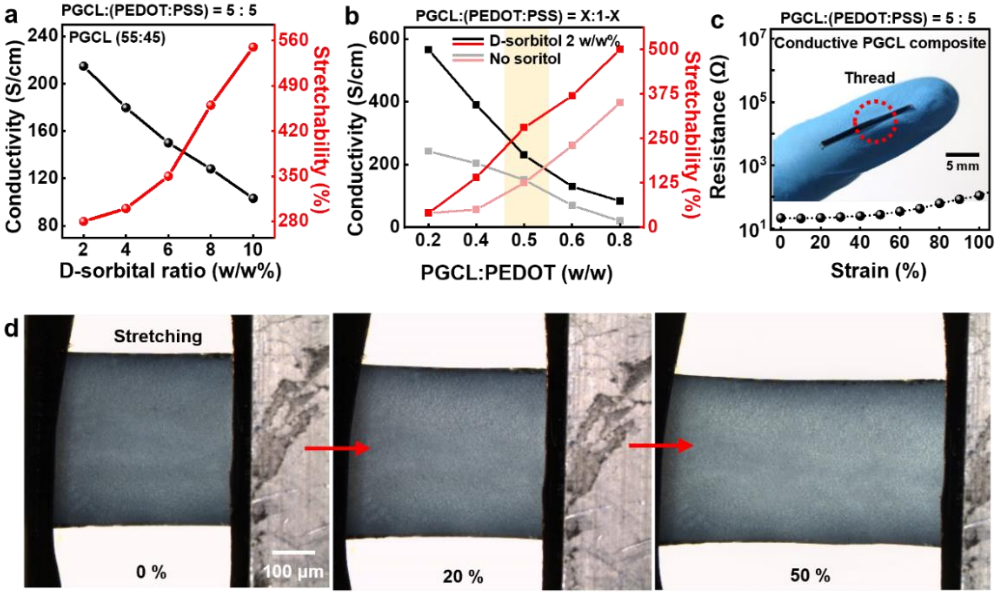

Fig. S8 Degradable conductive elastomer. **a** Addition effect with D-sorbitol ratio with respect to conductivity and stretchability in conductive composite at PGCL (55:45) : (PEDOT:PSS) = 5:5. **b** Evaluation of conductivity and stretchability with varied mixing ratio between PGCL and PEDOT:PSS. **c** Strain dependent resistance changes of the degradable conductive elastomeric thread. **d** Optical images of stretching degradable conductive elastomer applied with strain up to 50%

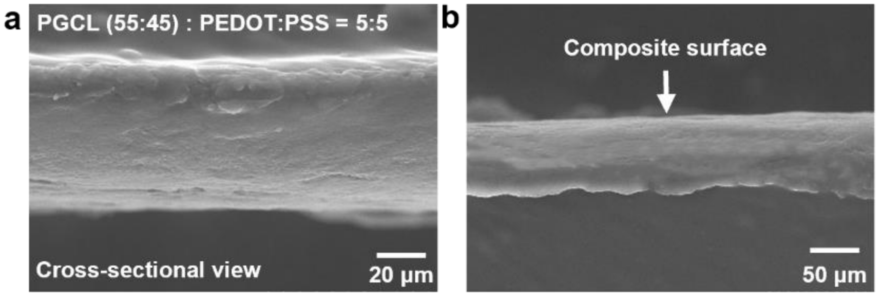

Fig. S9 Surface analysis of degradable conductive elastomer. **a** Cross-sectional SEM images of conductive composite and **b** flat surface image of the composite film

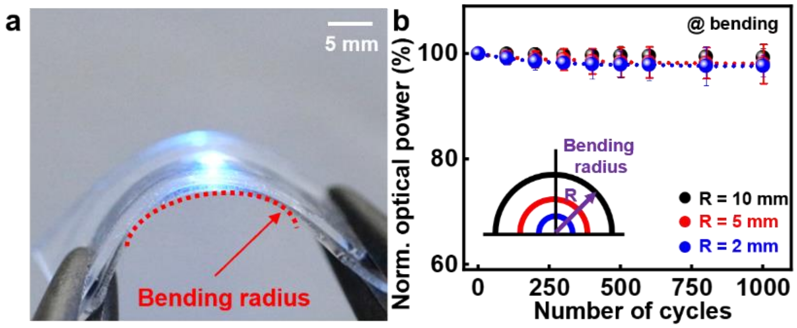

Fig. S10 Mechanical test of conductive composite. **a** Bending image of the conductive composite and **b** measured repeated bending test results to show changes in optical power of blue LED with varied bending radius

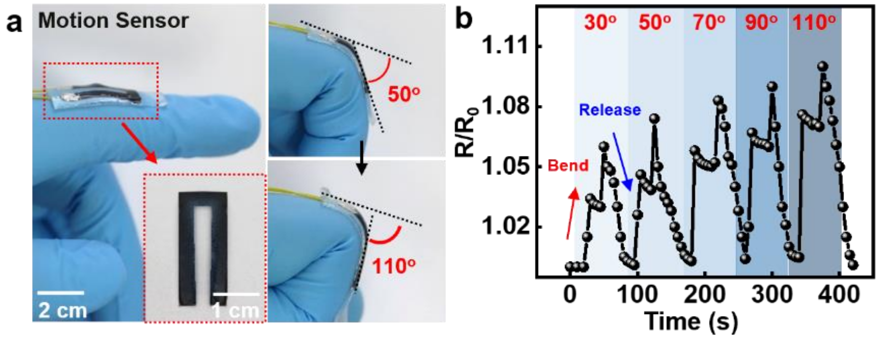

Fig. S11 Degradable Fig. motion sensor. **a** Strain sensing test of the attached device when flexing human's index finger with different bending angle. **b** Time-lapsed resistance profiles in response to diverse bending angle of human finger

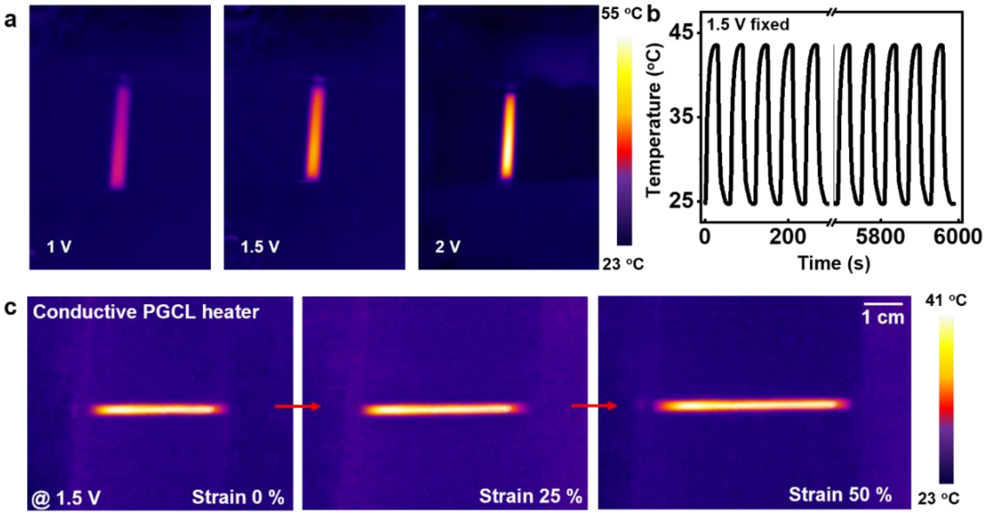

Fig. S12 Degradable elastic heating thread. **a** Infrared images of heating performance with different applying voltages. **b** Repetitive on/off test of heating thread. **c** Performance maintenance experiment in heating thread with mechanical deformation (strain, ~50%)

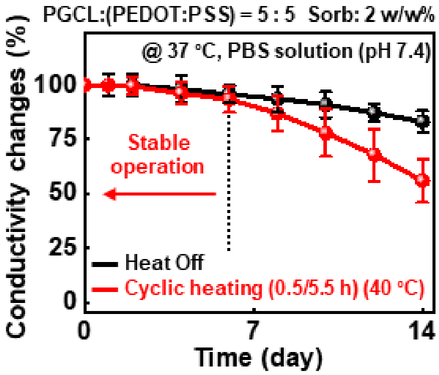

Fig. S13 Changes in conductivity of heating thread immersed in PBS solution at 37 °C Performance profile of heating thread w/o and w/ periodic heating for 2 weeks

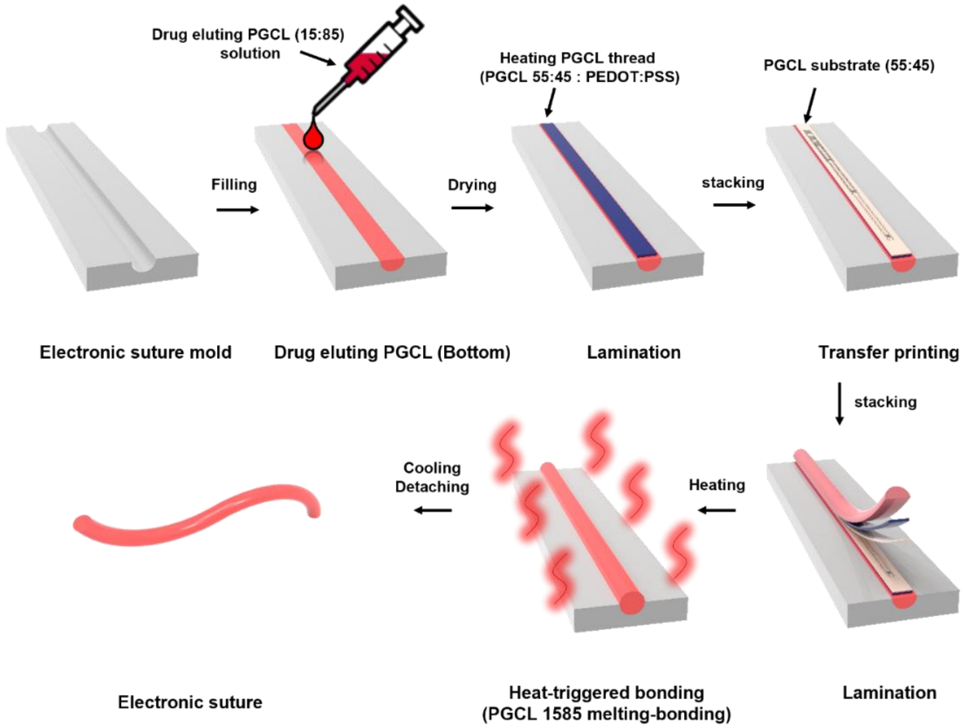

Fig. S14 Fabrication of medical electronic degradable suture. Schematic illustration of fabrication process of MED-suture

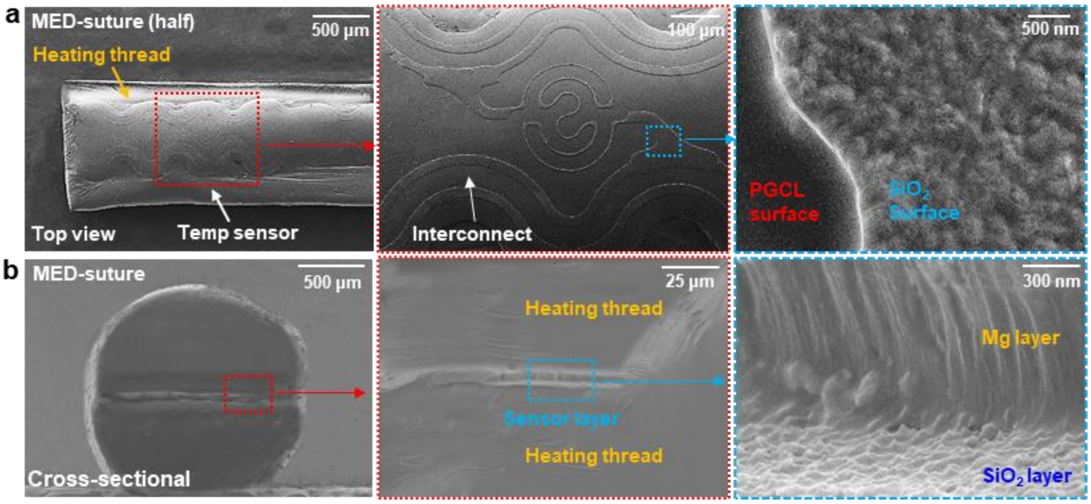

Fig. S15 Fabricated MED-suture. **a** SEM images showing microstructure of MED-suture half layer (top view) and **b** whole structure of MED-suture (cross-sectional view)

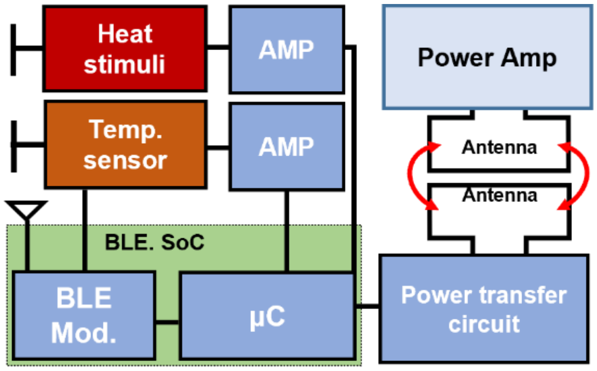

Fig. S16 Block diagram of wireless module. Circuit diagram for real-time monitoring, transmitting measured data and on-demand drug release

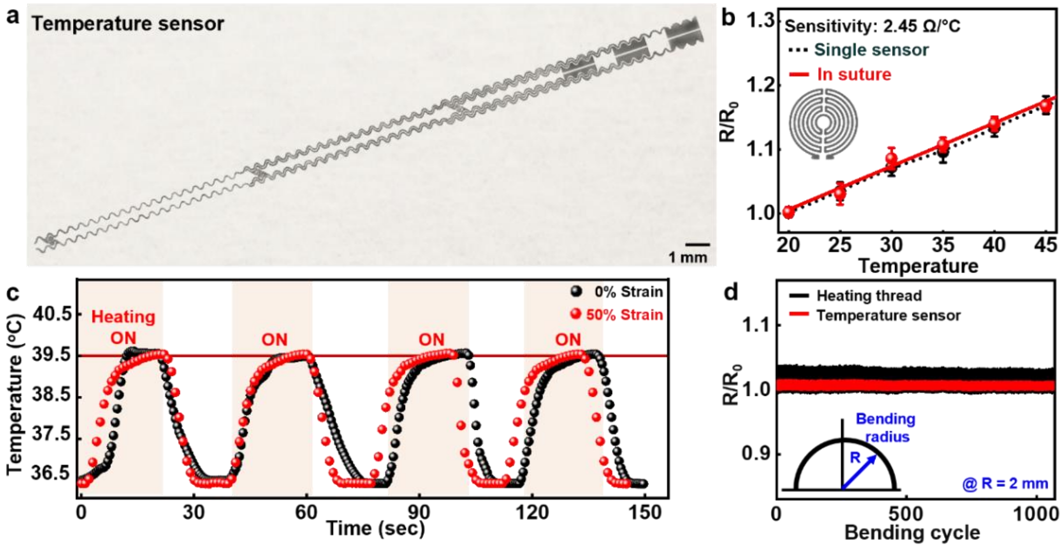

Fig. S17 Characterization of embedded temperature sensor and heater. **a** Optical image of temperature sensor of MED-suture. **b** Measured thermal sensitivity of temperature sensor between room temperature and body temperature. **c** Temperature profile in sensor of stable monitoring operation temperature of heat thread with deformation. **d** Reliable temperature sensor and heating thread performance even in repeated bending test of MED-suture

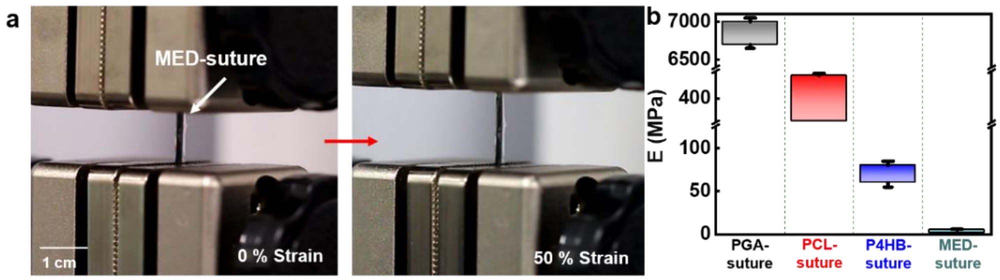

Fig. S18 Soft, elastic performance of MED-suture. **a** Mechanical tensile test images of MED-suture with 50% strain. **b** Comparative mechanical characteristics of commercialized absorbable sutures and MED-suture

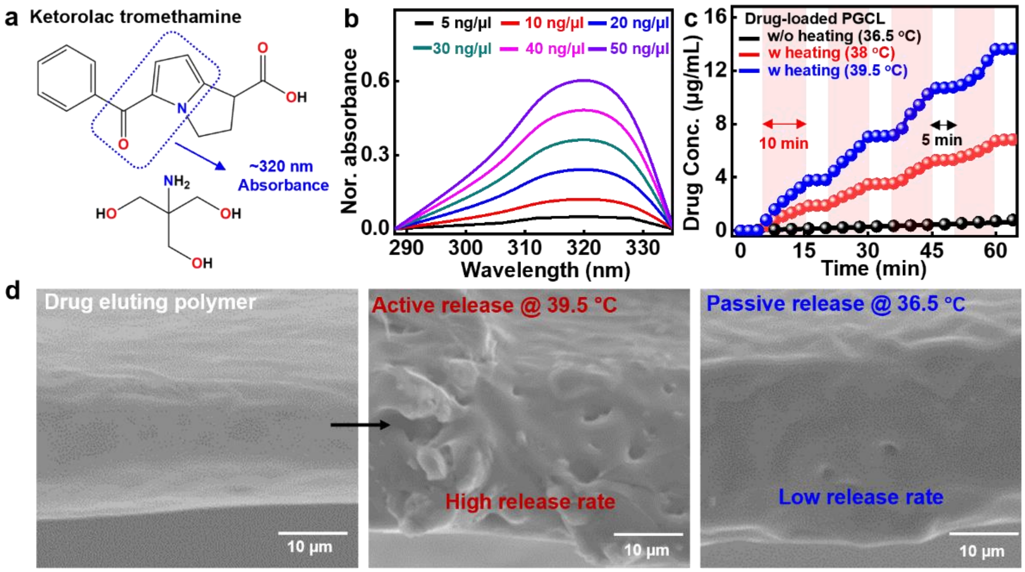

Fig. S19 Thermally induced drug release of MED-suture. **a** Molecular structure of ketorolac tromethamine as an anti-inflammatory agent. **b** Optical calibration of the drug concentration in the solution. **c** Drug release behaviors of drug loaded PGCL with different release conditions for 60 minutes. **d** Surface analysis of drug eluting layer after active and passive release

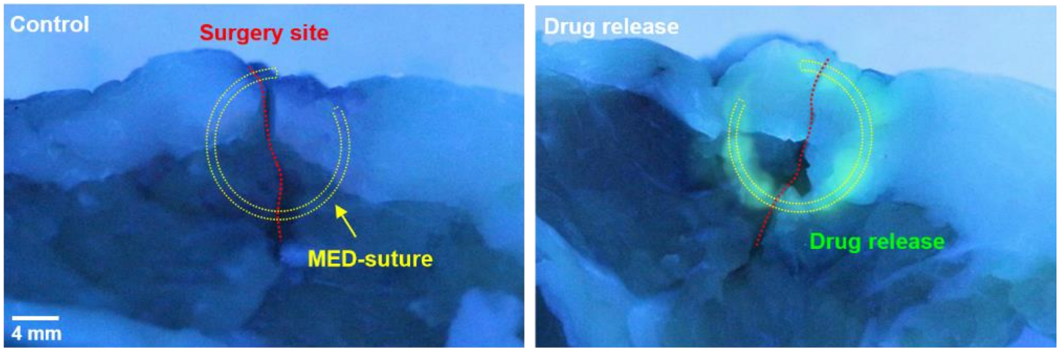

Fig. S20 *Ex vivo* results of controllable drug elution. Comparative resected porcine tissue images of control group (no thermal triggering) and repeated drug release group
